# Technological advancements and opportunities in Neuromarketing: a systematic review

**DOI:** 10.1186/s40708-020-00109-x

**Published:** 2020-09-21

**Authors:** Ferdousi Sabera Rawnaque, Khandoker Mahmudur Rahman, Syed Ferhat Anwar, Ravi Vaidyanathan, Tom Chau, Farhana Sarker, Khondaker Abdullah Al Mamun

**Affiliations:** 1 Advanced Intelligent Multidisciplinary Systems Lab, Institute of Advanced Research, United International University, Dhaka, Bangladesh; 2 School of Business and Economics, United International University, Dhaka, Bangladesh; 3 Institute of Business Administration, University of Dhaka, Dhaka, Bangladesh; 4 Department of Mechanical Engineering, Imperial College London, London, United Kingdom; 5 Institute of Biomaterials & Biomedical Engineering, University of Toronto, Toronto, Canada; 6 Department of Computer Science and Engineering, University of Liberal Arts Bangladesh, Dhaka, Bangladesh; 7 Department of Computer Science and Engineering, United International University, Dhaka, Bangladesh

**Keywords:** Neuromarketing, Neural recording, Machine learning algorithm, Brain computer interface, Marketing

## Abstract

Neuromarketing has become an academic and commercial area of interest, as the advancements in neural recording techniques and interpreting algorithms have made it an effective tool for recognizing the unspoken response of consumers to the marketing stimuli. This article presents the very first systematic review of the technological advancements in Neuromarketing field over the last 5 years. For this purpose, authors have selected and reviewed a total of 57 relevant literatures from valid databases which directly contribute to the Neuromarketing field with basic or empirical research findings. This review finds consumer goods as the prevalent marketing stimuli used in both product and promotion forms in these selected literatures. A trend of analyzing frontal and prefrontal alpha band signals is observed among the consumer emotion recognition-based experiments, which corresponds to frontal alpha asymmetry theory. The use of electroencephalogram (EEG) is found favorable by many researchers over functional magnetic resonance imaging (fMRI) in video advertisement-based Neuromarketing experiments, apparently due to its low cost and high time resolution advantages. Physiological response measuring techniques such as eye tracking, skin conductance recording, heart rate monitoring, and facial mapping have also been found in these empirical studies exclusively or in parallel with brain recordings. Alongside traditional filtering methods, independent component analysis (ICA) was found most commonly in artifact removal from neural signal. In consumer response prediction and classification, Artificial Neural Network (ANN), Support Vector Machine (SVM) and Linear Discriminant Analysis (LDA) have performed with the highest average accuracy among other machine learning algorithms used in these literatures. The authors hope, this review will assist the future researchers with vital information in the field of Neuromarketing for making novel contributions.

## Introduction

Neuromarketing, an application of the non-invasive brain–computer interface (BCI) technology, has emerged as an interdisciplinary bridge between neuroscience and marketing that has changed the perception of marketing research. Marketing is the channel between product and consumers which determines the ultimate sale. Without effective marketing, a good product fails to inform, engage and sustain its targeted audiences [1]. The expanding economy with new businesses is continuously evolving with changing consumer preferences. It is hard for the businesses to grow and sustain without having quantitative or qualitative assessment from their consumers. Newly launched products need even more effective marketing to successfully enter into a competitive market. However, traditional marketing renders only by posteriori analysis of consumer response. Conventional market research depends on surveys, focus group discussion, personal interviews, field trials and observations for collecting consumer feedback [2]. These approaches have the limitations of time requirement, high cost and unreliable information, which can often produce inaccurate results. In contrast to the traditional marketing research techniques, Neuromarketing allows capturing consumers’ unspoken cognitive and emotional response to various marketing stimuli and can forecast consumers’ purchase decisions.

Neuromarketing uses non-invasive brain signal recording techniques to directly measure the response of a customer’s brain to the marketing stimuli, superseding the traditional survey methods [3]. Functional magnetic resonance (fMRI), electroencephalography (EEG), magnetoencephalography (MEG), transcranial magnetic stimulator (TMS), positron emission tomography (PET), functional near-infrared spectroscopy (fNIRS) etc. are some examples of neural recording devices used in Neuromarketing research. By obtaining neuronal activity from the brain using these devices, one can explore the cognitive and emotional responses (i.e., like/dislike, approach/withdrawal) of a customer. Different stimuli trigger associated response in a human brain and the response can be tracked by monitoring the change in neuronal signals or brainwaves [4]. Further, the signal and image processing techniques and machine learning algorithms have enabled the researchers to measure, analyze and interpret the possible meanings of brainwaves. This opens a new door to detect, analyze and predict the buying behavior of customers in marketing research. Now with the help of brain–computer interface, the mental states of a customer, i.e., excitement, engagement, withdrawal, stress, etc., while experiencing a marketing stimuli can be captured [5]. Besides these brain signal recording techniques, Neuromarketing also utilizes physiological signals, i.e., eye tracking, heart rate and skin conductance measurements to gather the insight of audience’s physiological responses due to encountering stimuli. These neurophysiological signals with advanced spectral analysis and machine learning algorithms can now provide nearly accurate depiction of consumers’ preferences and likes/dislikes [6–8].

Early years of Neuromarketing generated a controversy between the academician and the marketers due to its high promises and lack of groundwork. From the claim of peeping into the consumer mind to finding the buy buttons of human brain, Neuromarketing has long been under the scrutiny of the academicians and researchers [9, 10]. However, academic research in this field has started to pile up and the scope of Neuromarketing to reveal and predict consumer behavior is gradually becoming evident. Neuromarketing Science and Business Association (NMSBA) was established in 2012 to bridge the gap between academicians and Neuromarketers, and it is promoting Neuromarketing research across the world with its annual event of Neuromarketing World Forum [11, 12]. It may be proposed that further dialogue may continue under such a platform for further industry–academia collaboration. Evidently, more than 150 consumer neuroscience companies are commercially operating across the globe and big brands (Google, Microsoft, Unilever, etc.) are using their insights to impact their consumers in a tailored and efficient way. Academic research, especially the high analytical accuracy from the engineering part of Neuromarketing has garnered this breakthrough and acceptance over the world. Hence, reviewing the building blocks of Neuromarketing is essential to evaluate its scopes and capacities, and to contribute new perspective in this field. Numerous literature reviews have been published focusing the theoretical aspect of consumer neuroscience, such as marketing, business ethics, management, psychology, consumer behavior, etc. [13–15]. However, systematic literature review from the engineering perspective with a focus on neural recording tools and interpretational methodologies used in this field is absent. In this regard, our article sets its premises to answer the following questions:

- What are the types of marketing stimuli currently being used in Neuromarketing?
- What are the brain regions activated by these marketing stimuli?
- What is the best brain signal recording tool currently being used in Neuromarketing research?
- How are these brain signals preprocessed for further analysis?
- And what are the current methods or techniques used to interpret these brain signals?

These questions will allow us to gain a comprehensive knowledge on the up-to-date research scopes and techniques in consumer neuroscience. After this brief introduction, our methodology of conducting this systematic review will be presented, followed by the state-of-the-art findings corresponding to the aforementioned questions and synthesis of the important results. We concluded this review with relevant inference from synthesized result and a recommendation for future researchers.

## Methodology

The systematic literature review is a process in which a body of literature is collected, screened, selected, reviewed and assessed with a pre-specified objective for the purpose of unbiased evidence collection and to reach an impartial conclusion [16]. Systematic review has the obligation to explicitly define its research question and to address inclusion–exclusion criteria for setting the scope of the investigation. After exhaustive search of existing literatures, articles should be selected based on their relevance, and the results of the selected studies must be synthesized and assessed critically to achieve clear conclusions [16].

In this systematic review, we would like to explore the marketing stimuli used in Neuromarketing research articles over the last 5 years with their triggered brain regions. We would also like to focus on the technological tools used to capture brain signals from these regions, and finally deliberate on signal processing and analytical methodologies used in these experiments.

Therefore, the inclusion criteria defined here are as follows:

- Literatures must be published in the field of Neuromarketing from 2015 to 2019.
- Studies must use brain–computer interface and/or other physiological signal recording device in their Neuromarketing experiments.
- Studies must have experimental findings from neural and/or biometric data used in Neuromarketing research.

The exclusion criteria for this review are set as:

- Any other literature review on Neuromarketing are excluded from this review.
- Book chapters are excluded from this review. Since Neuromarketing is comparatively a new research field, alongside relevant academic journal articles, book chapters conducting empirical experiments using BCI can only be included.
- Literatures written/published in any language other than English are excluded from this article.

To serve the purpose of this systematic literature review, a total of 931 articles were found across the internet by using the search item “Neuromarketing” and “Neuro-marketing” in valid databases. Among the screened publications, Table 1 presents the database source of selected 57 research articles including book chapters, which directly contribute to the Neuromarketing field with basic or empirical research findings.

**Table 1:** Number of articles found and selected

Name of the database	Results: search “Neuromarketing”	Results: search “Neuro-marketing”	Articles selected
Science direct	281	55	12
Wiley online	111	11	7
Emerald insight	115	8	14
IEEE	34	0	14
Sage	12	15	6
Taylor Francis online	106	36	4
	Total found: 806	Total found: 125	Total selected: 57

As for the aggregation of relevant existing literatures, the researchers defined that the search for articles would be performed in six databases—Science Direct, Emerald Insight, Sage, IEEE Xplore, Wiley Online Library, and Taylor Francis Online. After the initial article accumulation, the articles were exhaustively screened by the authors by reviewing their title, abstract, keywords and scope to match the objective of this research. Once the studies met our aforementioned inclusion criteria, they were selected for further review and critical analysis. Table 2 classifies the selected articles in terms of the aforementioned dimensions.

**Table 2:** Studies selected on the dimensions of this review

Dimensions		Published articles
i. Marketing stimuli used in Neuromarketing	Product	Chew et al. [17], Yadava et al. [18], Rojas et al. [19], Pozharliev [20], Touchette and Lee [21], Marques et al. [22], Shen et al. [23], Çakir et al. [24], Hubert et al. [25], Hsu and Chen et al. [26], Hoefer et al. [27], Gurbuj and Toga [28], Wriessnegger et al. [29], Wang et al. [30], Wolfe et al. [31], Bosshard et al. [32], Fehse et al. [33].
	Price	Çakar et al. [34], Marques et al. [22], Çakir et al. [24], Gong et al. [35], Pilelienė and Grigaliūnaitė [36], Hsu and Chen [26], Boccia et al. [37], Venkatraman et al. [38], Baldo et al. [39].
	Promotion	Soria Morillo et al. [40], Yang et al. [41], Cherubino et al. [42], Soria Morillo et al. [43], Vasiljević et al. [44], Yang et al. [45], Pilelienė and Grigaliūnaitė [36], Daugherty et al. [46], Royo et al. [47], Etzold et al. [48], Chen et al. [49], Casado-Aranda et al. [50], Randolph and Pierquet [51], Nomura and Mitsukura [52], Ungureanu et al. [53], Goyal and Singh [54], Oon et al. [55], Singh et al. [56].
ii. Activation of brain region due to marketing stimuli		Soria Morillo et al. [40], Chew et al. [17], Cherubino et al. [42], Soria Morillo et al. [43], Çakar et al. [34], Boksem and Smitds [57], Bhardwaj et al. [58], Venkatraman et al. [38], Touchette and Lee [21], Yang et al. [45], Marques et al. [22], Gong et al. [35], Gordon et al. [59], Krampe et al. [60], Hubert et al. [25], Çakir et al. [24], Holst and Henseler [61], Hsu and Cheng [62], Hoefer et al. [27], Chen et al. [49], Casado-Aranda et al. [50], Wang et al. [30], Jain et al. [63], Wolfe et al. [31], Bosshard et al. [32], Fehse et al. [33].
iii. Neural response recording techniques	EEG	Soria Morillo et al. [40], Yang et al. [41], Chew et al. [17], Cherubino et al. [42], Soria Morillo et al. [43], Yadava et al. [18], Doborjeh et al. [64], Çakar et al. [34], Kaur et al. [65], Baldo et al. [19], Boksem and Smitds [57], Pozharliev et al. [20], Venkatraman [38], Touchette and Lee [21], Yang et al. [45], Pilelienė and Grigaliūnaitė [36], Shen et al. [23], Daugherty et al. [46], Royo et al. [47], Gong et al. [35], Gordon et al. [59], Hsu and Chen et al. [26], Hoefer et al. [27], Randolph and Pierquet [51], Nomura and Mitsukura [52], Bhardwaj et al. [58], Fan and Touyama [66], Rakshit and Lahiri [67], Jain et al. [63],Ogino and Mitsukura [68], Oon et al. [55], Bosshard et al. [32].
	fMRI	Venkatraman et al. [38], Marques et al. [22], Hubert et al. [25], Hsu and Cheng [62], Chen et al. [49], Casado-Aranda et al. [50], Wang et al. [30], Wolfe et al. [31], Fehse et al. [33].
	fNIRS	Çakir et al. [24], Krampe et al. [60].
	EMG	Missagila et al. [69]
	Eye tracking	Venkatraman [38], Rojas et al. [19], Pilelienė and Grigaliūnaitė [36], Çakar et al. [34], Ceravolo et al. [70], Ungureanu et al. [53]
	Galvanic skin response, heart rate	Cherubino et al. [42], Çakar et al. [34], Magdin et al. [71], Goyal and Singh [54], Singh et al. [56].
iv. Brain signal processing in Neuromarketing		Cherubino et al. [42], Bhardwaj et al. [53], Venkatraman [38], Pozharliev et al. [20], Boksem and Smitds [57], Wriessnegger et al. [29], Fan and Touyama [66], Pilelienė and Grigaliūnaitė [36], Yadava et al. [18], Baldo et al. [19], Clerico et al. [72], Chen et al. [49], Casado-Aranda et al. [50], Hsu and Cheng [62], Taqwa et al. [73], Bhardwaj et al. [58],Wang et al. [30], Rakshit and Lahiri [67], Goyal and Singh [54], Jain et al. [63], Oon et al. [55], Fehse et al. [33],
v. Machine learning applications in Neuromarketing		Soria Morillo et al. [40], Yang et al. [41], Chew et al. [17], Soria Morillo et al. [43], Yadava et al. [18], Doborjeh et al. [64], Gordon [59], Gurbuj and Toga [28], Wriessnegger et al. [29], Wang et al. [30], Taqwa et al. [73], Bhardwaj et al. [58], Randolph and Pierquet [51], Fan and Touyama [66], Rakshit and Lahiri [67], Goyal and Singh [54], Jain et al. [63], Ogino and Mitsukura [68], Oon et al. [55], Singh et al. [56].

By exploring the articles selected to develop this systematic review, it was possible to successfully categorize the trends and advancements in Neuromarketing field in following dimensions:

i. Marketing stimuli used in Neuromarketing research
ii. Activation of the brain regions due to marketing stimuli
iii. Neural response recording techniques
iv. Brain signal processing in Neuromarketing
v. Machine learning applications in Neuromarketing.

Some of these Neuromarketing studies have used eye tracking, heart rate, galvanic skin response, facial action coding, etc., with or without brain signal recording techniques to gauge the consumer’s hidden response. As they are the response from autonomous nervous system (ANS), they have proven themselves as successful means of exploring consumer’s focus, arousal, attention and withdrawal actions. Hence, this study includes articles those empirically used these tools to answer Neuromarketing questions, since this study mainly focuses on the engineering perspective. Interpreting the neural data with only statistical analysis has been out of scope of this paper.

## Systematic review on the advancements of Neuromarketing

Neuromarketing research utilizes marketing strategies in the form of stimuli, and aims to invoke, capture and analyze activities occurring in different brain regions while subjects experience these stimuli. To conduct a systematic review on this matter, it is important to recall the interconnection between brain functions with human behavior and actions triggered by the external stimuli. The knowledge of brain anatomy and the physiological functions of brain areas as well as the physiological response due to external stimuli along with it, makes it possible to model brain activity and predict hidden response. For this purpose, current neural imaging systems and neural recording systems have contributed much to capture the true essence of consumer preferences. This section will discuss the marketing stimuli, their targeted brain regions, neural and physiological signal capturing technologies used over the last 5 years in Neuromarketing research. Comparing these signals with their associated anatomical functionality some studies have already reached high accuracy. A number of the selected studies have used machine learning techniques to predict like/dislike and possible preference from the test subjects.

For the purpose of Neuromarketing experiments, the following literatures selected right-handed participants, with normal or corrected-to-normal vision, free of central nervous system influencing medications and with no history of neuropathology.

### Marketing stimuli used in Neuromarketing

As Neuromarketing is a focus of marketers and consumer behavior researchers, different strategies from marketing have been applied in Neuromarketing and they are being investigated for quantitative assessment from neurological data. Nemorin et al. asserts that Neuromarketing differentiates from any other marketing models as it bypasses the thinking procedures of consumers and directly enters their brain [74]. Over the last 5 years, Neuromarketing stimuli has been mainly in two forms—products with/without price, and promotions. Product can be defined as physical object or service that meets the consumer demand. In Neuromarketing, product can be physical such as tasting a beverage to conceptual like a 3D (three dimensional) image of the product. Price in Neuromarketing experiments is mostly seen as a stimuli is most of the time intermingled with product or promotion. However, it plays an important role that determines the decision of test subjects to buy or not to buy the product [75].

Consumer response to a product has been recognized by either physically experiencing the product or by visualizing the image of it. To understand the user esthetics of 3D shapes, Chew et al. [17], used virtual 3D bracelet shapes in motion and recorded the brain response of test subjects with EEG with motion. As 3D visualization of objects for preference recognition is a new area of research, the authors used mathematical model (Gielis *superformula*) to create 3D bracelet-like objects. Their study displayed 3D shapes appear like bracelets as the product to subjects. Using the 3D shapes gave the authors an advantage to produce as many of 60 bracelet shapes to conduct the research on. Another new product was the E-commerce products presented to the test subjects by Yadava et al. and Çakar et al. [18, 34]. Yadava et al. proposed a predictive modeling framework to understand consumer choice towards E-commerce products in terms of “likes” and “dislikes” by analyzing EEG signals. In showing E-commerce product, they showed a total of 42 product images to the test participants. These product images were mainly of apparels and accessory items such as shirts, sweaters, shoes, school bags, wrist watches, etc. The test participants were asked to disclose their preference in terms of likes and dislikes after viewing the items [18]. Çakar et al. used both product and price to explore the experience during product search of first-time buyers in E-commerce. To motivate the participants, this research provided each participants around 73 USD as a gift card to use during the experiment. The test participants were asked to search and select three products of their interest from an e-commerce website and reach the maximum of their gift card limit to activate. Test subjects often experienced negative emotion while being unable to find necessary buttons such as “add to cart” or “sorting options” [34]. These Neuromarketing experiments on E-commerce products may help developers to build better user experience. Retail businesses lose large amount of money when they invest in the wrong product. Among retail products, shoes have thousands of blueprints for manufacturing. Producing thousands of shoes of different designs to satisfy consumers can be laborious and unprofitable since a large number of the designs turn out to be failures. Baldo et al. directly used 30 existing image of shoe designs to show the test subjects to and to choose from a mock shop showing on the screen [39]. EEG signals were recorded during the whole shoe selection time and then subjects were asked to rate the shoes in a rank of 1 to 5 of Likert scale. This experiment helped realize brain response-based prediction can supersede self-report-based methods, as the simulation on sales data showed 12.1% profit growth for survey-based prediction, and 36.4% profit growth for the brain response-based prediction.

Similar to the shoe experiment, Touchette and Lee [21] experimented on the choice of apparel products among young adults, based on Davidson’s frontal asymmetry theory. EEG signals were recorded while 34 college students viewed three attractive and three unattractive apparel products on a high-resolution computer screen in a random order. Pozharliev et al. [20] experimented on the emotion associated with visualizing luxury brand products vs. regular brand products. The experiment displayed 60 luxury items and 60 basic brand items to 40 female undergraduate students to recognize the brain response of seeing high emotional value (luxury) products in social vs. alone atmosphere. The study found that, luxury brand products invoked a higher emotional value in social atmosphere which could be utilized by the marketers. Bosshard et al. and Fehse et al. experimented on brand images and the comparison between the brain responses associated with preferred and not preferred brands [32, 33]. In the study performed by Bosshard et al., consumer attitude towards established brand names were measured via electroencephalography. Subjects were shown 120 brand names in capital white letter in Tahoma font on black background and without any logo while their brain responses were recorded. On the other hand, Fehse et al. compared the brain response of test subjects while they visualized blocks of popular vs. organic food brand logos. These experiments on brand image may help marketers to recognize the implicit response of consumers on different types of branding.

As price is mentioned as an important factor that determines the user’s interest on purchasing a product, a number of Neuromarketing studies have used price alongside the products. In the aforementioned study by Çakar et al. [34] price was displayed while recording brain response during first-time e-commerce user experience. Marques et al. [22], Çakir et al. [24], Gong et al. [35], Pilelienė and Grigaliūnaitė [36], Hsu and Chen [26], Boccia et al. [37], Venkatraman et al. [38], and Baldo et al. [39] have included price as a marketing stimuli with the product or promotional.

An interesting concept was tried by Boccia et al. to recognize the relation between corporate social responsibilities and consumer behavior. The author attempted to identify if consumers were willing to pay more for the products from socially or environmentally responsible company. Consumers were found to prefer the conventional companies over the socially responsible companies due to lesser price. Marques et al. [22] investigated the influence of price to compare national brand vs. own-labeled branded products. In the experiment of Çakir et al, product then product and price were shown to the subjects before decision-making time and the brain responses were recorded through fNIRS [24]. Sometimes price can play a passive role in the form of discounts or gifts in a promotional. Gong et al. innovatively designed an experiment to compare consumer brain response associated with promotional using discount (25% off) vs. gift-giving (gift value equivalent to the discount) marketing strategies. Their study found that lower degree of ambiguity (e.g., discounts) better motivates consumer decision-making [35]. Hsu and Chen used price as a control variable in their wine tasting experiment. As price plays a pivotal role in purchase decision, two wines were selected of approximately equal price $15. Then the EEG signals of test subjects were recorded during the wine tasting session [26].

Promotion is the communication from the marketers’ end to influence the purchase decision of consumers [75]. In Neuromarketing research, promotion is usually found as the TV commercials and short movies for advertisement. One of the key focus of Neuromarketers is to evaluate the consumer engagement of advertisements. Predicting the engagement of advertisements before broadcasting them on air, ensures higher rate of successful promotions.

In 2015, Yang et al. used six smartphone commercials of different brands to compare among them in terms of extract cognitive neurophysiological indices such as happiness, surprise, and attention as well as behavioral indices (memory rate, preference, etc.) [41]. A common experimental design procedure is found among the promotion-based Neuromarketing experiments, that is subjects are first made comfortable in the experimental setting, consecutive advertisements were placed at a time distance no shorter than 10 s and consecutive advertisements used neutral stimuli such as white screen, green scenario, blank in between them to stabilize the test participants.

The Neuromarketing experiments of Soria Morillo et al. [40, 43] tried to find out the electrical activity of audience brain while viewing advertisement relevant to audiences’ taste. They display used 14 TV commercials displayed to their 10 test subjects for their experiment and predicted like or dislike response from audience with the help of advanced algorithms. Cherubino et al. [42] investigated cognitive and emotional changes of cerebral activity during the observation of TV commercials among different aged population. Among seven TV commercials displayed during the experiment, one commercial with strong images was analyzed for the adults’ and older adults’ reaction. Other than them, Vasiljević et al. [44] used Nestle advertisement to measure consumer attention though pulse analysis; Daugherty et al. [46] replicated an experiment of Krugman (1971) using both TV advertisements and print media advertisements to recognize how consumers look and think; Royo et al. [47] focused on consumer response while viewing advertisements of sustainable product designs. For their experiment, an animated commercial was made containing verbal narrative of sustainable product and an existing commercial was used to convey the visual narrative of conventional product. Venkatraman et al. focused on measuring the success of TV advertisements using neuroimaging and biometric data [38]. Randolph and Pierquet [51] showed super bowl commercials to undergraduate students to compare the class rank of the commercials and the neural response from the test subjects. Nomura and Mitsukura [52] identified emotional states of audiences while watching favorable vs. unfavorable TV commercials. They selected 100 TV commercials among which 50 commercials were award winning which were labeled as favorable advertisements. Singh et al. [56] used promotion in the form of static vs. video advertisements to predict the success of omnichannel marketing strategies. Ungureanu et al. [53] measured user attention and arousal by eye tracking while surfing through web page containing static advertisements, while Goyal and Singh [54] utilized facial biometric sensors to model an automated review systems for video advertisements. Oon et al. [55] used merchandise product advertisement clips to recognize user preference. Singh et al. [56] used video advertisements to measure visual attentions of audiences.

Most of the TVC (television commercials) in these literatures had a standard time of 30 s. In Neuromarketing, these TVCs were displayed in between other videos such as documentary film, gaming video, drama, etc., to capture the true response of consumers.

Sometimes Neuromarketing is observed dealing with advertisement of different purposes, such as social advertisements or gender-related advertisements. The application of Neuromarketing in social advertisement is to predict the success of these ads to reach its messages to the targeted social groups [45, 49, 69]. Chen et al. [49] experimented on the neural response of adolescent audiences while they are exposed to e-cigarette commercials. Another social advertisement stimuli of smoking cessation frames was used by Yang [45], to understand what types of frames (positive/negative) achieve better attention from smokers and non-smokers. Gender plays a substantial role in advertisement industry from celebrity endorsement to gender-targeted marketing. Missaglia et al. [69] conducted a research on fast marketed consumer goods (FMCG) advertisements with celebrity vs. non-celebrity female spokesperson. Casado-Aranda et al. [50] worked on gender-targeted advertisements using congruent vs. incongruent product–voice combination. These studies show us the diversity of marketing stimuli for future Neuromarketing applications.

### Activation of brain regions due to marketing stimuli

Human brain is a matter of profound astonishment. The anatomical development of our brain resulted in the complex web of cognitive and emotional process we experience every day. The evolution of vertebrate brain was initially proposed by Paul D. MacLean in his Triune Brain model [76]. In his hypothesis, evolution of vertebrate brain is formed through three phases. First the reptilian complex, which indicates the association of instincts with the anatomical structure basal ganglia. The paleomammalian complex consists of septum, amygdalae, hypothalamus, hippocampal complex, and cingulate cortex as the limbic system. These organelles were associated with motivation and emotional response of mammalian brain. Finally, neomammalian complex consists of cerebral neocortex or the outer layer of advanced mammalian brain, which is particularly a unique feature of human brain. In the cerebral neocortex, we find four lobes which control our sensory, motor, emotional and cognitive processes [76]. The triune brain model has been rejected by new neuroscientists due to the interconnectivity of human brain structures and their function. However, the anatomical structure of human brain explained by this theory plays a vital role in recognizing cognitive, emotional and behavioral process.

Understanding the anatomy of human brain has showed itself indispensable in Neuromarketing research, as its functionality is deeply associated with the interpretation of neural response. The outer layer of the human brain is a complex system organized in four lobes, namely (frontal, parietal, temporal and occipital lobes), each having distinct functionalities for cognitive, emotional, and motor responses. The frontal lobe is the region where most of our thoughts and conscious decisions are made [77]. Cognitive decision-making mainly takes part in the prefrontal region of this lobe, and movement-related decisions are made in the end part of frontal lobe. Information about taste, touch and movement is processed by the parietal lobe. The occipital lobe is the primary center for visual processing, and the temporal lobe is responsible for visual memories, auditory recognition and integrating new sensory information with memories [78]. Besides the primary lobes, cerebral cortex brain anatomy has gyri and sulci which create the folded appearance of the brain. The gyri functions on increasing surface area for information processing. Alongside the primary lobes, gyri of these lobes can be considered as the region of interest (ROI) in neural imaging techniques [79].

Deeper structures of the human brain consisting thalamus, amygdalae, etc., produces sensory and instinctual responses which are later transported to the cerebral cortex. Hypothalamus works as the master control of our autonomic system. Sleep, hunger, thirst, blood pressure, body temperature, sexual arousal are controlled and regulated by hypothalamus. Thalamus on the other hand regulates sensory information, attention and memory. Amygdalae originate our emotional response and hippocampus is the mainframe of our memory [77].

Retrieving information from brain requires diverse types of methodology. In Neuromarketing experiments, different parts of brain are selected for retrieving distinct information. An experiment which solely focuses on attention might only look at the signals from frontal lobe, whereas experiments focusing on buyer’s motivation might want to look at deeper structures [38].

According to Soria Morillo et al., brain signal acquisition may capture neural signals either from cerebral cortex or from the deeper layer of the brain [40, 43]. Their experiment on TV advertisement liking recognition initially uses information only from prefrontal cortex using a single electrode EEG device. Their experiment showed, it is possible to classify like/dislike with information collected solely from frontal lobe.

Similarly, Cherubino et al. emphasized on the significance of frontal cortex (FC) and prefrontal cortex (PFC) in Neuromarketing studies. PFC processes the conscious and unconscious cognitive and emotional information. Hence, devices using only a single sensor select PFC as their signal acquisition region [42]. Also, ventromedial prefrontal cortex corresponds to motivational behaviors, imaging of which by fMRI or MEG can reveal purchase motivations [22].

Neural communication in the brain is conducted through the action potentials, or the firing of neurons [80]. A neuronal signal is the electrochemical information that neurons send to each other. These information are acquired as signals of non-linear pattern called the brainwaves [80]. These brainwaves are further associated with the neural signature of brain states. The neural signature is divided into frequency bands known as rhythms, such as the delta (0.1–4 Hz), theta (4–8 Hz), alpha (8–12 Hz), beta (12–30 Hz), and gamma (30–90 Hz). These frequency bands are related to different brain states, regions, functions or pathologies. Delta (*δ*) waves are characteristic of deep sleep and have not been explored for BCI applications [81]. Theta (*θ*) waves are enhanced during sleep in adults and often related to various brain disorders. During wakefulness under relaxed conditions alpha (*α*) waves with moderate amplitude appear spontaneously. Beta (*β*) waves have less amplitude and are strongly related to motor control and engagement or decision-making procedure. Gamma (*γ*) waves are associated with movement-related activity of the brain and intensely observed in invasive neural recording [81].

In Neuromarketing, beta wave amplitudes are associated with reward processing which can further predict success of a product or TVC (Boksem and Smitds) [57].

Frontal alpha asymmetry is a key concept of hemisphere-based like–dislike classification approach. When the brain is considered as two hemispheres, left and right frontal cortices show hemispheric asymmetry in their activation during processing positive and negative emotion. Another term for emotional engagement, Approach–Withdrawal Index refers to the emotional response from Frontal Alpha Asymmetry theory [34]. Frontal Asymmetry Index is a marker of approach and avoidance. “Emotional Engagement” in Neuromarketing is expressed as the power of specific frequency bands from left and right frontal regions. The F3/F4 and F7/F8 electrodes are the best candidates for these EEG power reception as they are positioned at the most sensitive places (International 10–20 System). The alpha frequency band (8–12 Hz) is commonly used in the frontal alpha asymmetry theory. However, as the alpha activity corresponds with relaxation and meditation, it is negatively correlated with cognitive engagement.

Frontal Asymmetry Index is measured from the equation:

$${\text{Frontal Asymmetry Index = ln }}\frac{\text{Alpha Power of Right F4 or F8}}{\text{Alpha Power of Left F3 or F7}}.$$

Higher the Frontal Asymmetry Index value, the more approach response is obtained from the test subjects and vice versa. This high or positive asymmetry score can determine pleasant feeling of a test subject and vice versa, which was explored in the study conducted by Touchette and Lee [21].

Neuroimaging and neural signal recording devices use these locations and brain states to map the mind of a consumer. A standard 10–20 system has been established, which is an internationally recognized method to apply the EEG sensors or electrodes on a human scalp. EEG electrodes under 10–20 system have letters to express their location on skull such as prefrontal (Fp), frontal (F), temporal (T), parietal (P), occipital (O), and central (C). Even number of electrodes are placed on the right side of the head.

On the other hand, a test subject is placed inside an fMRI machine where the activities of the cortices can be recorded from the hemodynamic response or blood oxygen level-dependent (BOLD) imaging process. fMRI can look deeper within the spatial range from millimeters to centimeters. This enables Neuromarketing researchers using fMRI imaging to examine the response at putamen, thalamus, amygdalae and even in the hippocampus.

Functional near-infrared spectroscopy (fNIRS) is another new brain imaging tool which uses the hemodynamic responses associated with neuronal activities [24, 60]. However, fNIRS has a lower spatial resolution than fMRI and cannot look deeper than 4 cm.

Alongside brain regions associated with neural response, the human has a peripheral system which corresponds to cognitive and emotional processes. Eye movement, skin conductance, heart rate, facial expression all are result of neural processes. Eye tracking is primarily considered as the physiological response in consumer neuroscience, however studies have suggested eye tracking as a result of activation of the visual cortex or a secondary neural response [34, 36, 38, 53, 70].

Neuromarketing experiments focused on the affect–circumflex coordinate or valance–arousal coordinate use autonomic nervous system (ANS) response from sweat glands of hands or galvanic skin response (GSR), and cardiovascular measure or heart rate (HR). GSR is viewed as a sensitive and convenient measure for indexing changes in sympathetic arousal associated with emotion, cognition and attention. On the other hand, HR correlates with the emotional valence of a stimulus, e.g., the positive or negative component of the emotion [34].

Considering the available regions to capture signals from, it is highly likely that Neuromarketing will exponentially improve its recognition and predictions in user response and preferences.

### Neural response recording techniques

The groundwork in Neuromarketing field is evidently indebted to the advancement of neuroimaging and neural recording tools. Neurophysiological tools, such as electroencephalography (EEG), functional magnetic resonance imaging (fMRI), eye tracking, skin conductance, heart rate, etc., made it feasible to conduct the academic and commercial Neuromarketing research. Many research-grade neurophysiological and biometric signal capturing devices are now available in the market. However, some devices have cost and mobility advantages over the others and therefore replacing the expensive and immobile devices for Neuromarketing purpose.

Among all neuroimaging devices, functional magnetic resonance imaging (fMRI) has been the most widely used neuroimaging technique in Neuromarketing research during the initial time of consumer neuroscience. The reason behind the wide acceptance of fMRI is that it offers the identification of cerebral regions associated with cognitive and emotional process. Combining magnetic field and radio waves, fMRI produces a sequence of images of the cerebral activity by measuring the blood flow of the cerebral cortex [38]. The signal imaged in fMRI is called BOLD (blood oxygen level dependent) signal. This technology also allows 3D views of the coordinates that denote certain location, making possible to investigate deeper brain structures [57]. The primary disadvantages of this method are that it is very expensive and till now has a poor temporal resolution. The computer screen used in fMRI refreshes the image every 2 to 5 s. This low temporal resolution to the order of seconds due to the time requirement of the cerebral blood flow’s increment after being exposed to the stimuli, makes fMRI unsuitable for tracking brain activities to the order of milliseconds, which is required in video advertisement analysis. Other disadvantage is the head of the subject must remain static during the whole image recording process [62]. This restriction causes complex preprocessing and movement-related artifact removal from the fMRI signals. A number of studies, i.e., Venkatraman et al. [38], Marques et al. [22], Hubert et al. [25], Hsu and Cheng [26], Chen et al. [49], Casado-Aranda et al. [50], Wang et al. [30], Wolfe et al. [31], Fehse et al. [33], etc., have used fMRI as the neuroimaging technique in their Neuromarketing studies. fMRI in all studies required the test subjects to remain static and displayed the subjects the images and commercials of products for 3–5 s. Later the subjects had to make purchase decision within 5 s after their exposure to the stimuli [50]. Researchers over the last 5 years are found using 3-T fMRI scanner 3.0-T Siemens Magnetom Trio system MRI Scanner equipped with a 32-channel bridge head coil (Hubert and Hsu and Cheng) [25, 62] and 3 Tesla Siemens Verio scanner (Wang et al. [30]). Cost of an fMRI machine can be from $500,000 to $3 million varying on its spatiotemporal resolution.

Alongside fMRI, electroencephalography (EEG) is another popular tool used in Neuromarketing research. Number of research in Neuromarketing using EEG devices is increasing due to EEG’s cost efficiency high temporal resolution and mobility advantages. The EEG measures electrical activity in the cerebral cortex, the outer layer of the brain. EEG devices are placed following the 10–20 system. According to the 10–20 system, the 10 and 20 refer to the actual percentage of distances between adjacent electrodes which are either 10% or 20% of the total front–back or right–left distance of the skull [82]. As EEG is portable and allows capturing signal from cerebral cortex with high temporal resolution, it is mainly used in TV commercial engagement or success analysis. EEG signal of interest in Neuromarketing are mainly event-related potential (ERP), and late positive potential (LPP). ERP and LPP are used by Pozharliev et al. [20] to measure the emotional value of luxury products. Çakar et al. [34] used ERP to explore the experience of first-time user of E-commerce product. Pilelienė and Grigaliūnaitė [36]) used ERP along with eye tracking signal to measure the impact of celebrity spokesman in TVC. Shen et al. [23] used ERP and LPP to explore the influence of rating reviews on online products.

Research-grade EEG devices are vastly used in Neuromarketing. Emotiv Epoc and Emotive Epoc+ were found as the mostly commonly used EEG devices in the review. These devices were used in the studies of Yang et al. [45], Chew et al. [17], Soria Morillo et al. [40], Yadava et al. [18], Royo et al. [47], Jain et al. [63], and Singh et al. [56]. Emotive Epoc+ is a moveable, cost-effective EEG headset having 14 electrodes those cover the frontal, temporal, parietal and occipital lobes with channels AF3, F7, F3, FC5, T7, P7, O1, O2, P8, T8, FC6, F4, F8, AF4. The acquired brain signals from Emotiv Epoc+ are highly dependable and have already been used in these scientific researches. Another popular EEG device in Neuromarketing, NeuroSky Mindwave, has only one sensor placed on the prefrontal cortex of the head or the forehead. Unlike EEG devices with wet electrodes, Neurosky Mindwave employs a biosensor which does not require any conductive medium to be applied on the test subject’s scalp [40]. With the help of NeroSkyLab, the provided scientific research tool, data viewing and analysis can be conducted easily by non-engineer population. In 2015, Soria Morillo et al. and Ogino and Mitsukura in 2018 conducted Neuromarketing experiment with NeuroSky device and with the help of machine learning algorithm, their choice prediction accuracy was over 70% [40, 68]. A 10-channel EEG device BrainAmp, from BrainProducts GmBh was used in the Neuromarketing experiment conducted by Cherubino et al. [42]. Another device EEGO Sports from ANT Neuro (32 channels) was used to analyze non-linear features of EEG signals by Oon et al. [55]. B-alert X10 headset from ABM consisting 9 electrode channels is found in use by the experiment of Chew et al. [17]. 8-channel E-Prime from Neuroscan is another EEG device is used in the sales strategy experiment by Gong et al. and Touchette et al. conducted their apparel liking experiment with NeXus-10 biofeedback system. EEG devices have different sampling rates starting from 128 to 512 Hz. This sampling rate determines the highest frequency recordable by the EEG device. In general EEG has a lower frequency spectrum, having Gamma band up to 90 Hz. This gives researchers advantage to choose the right EEG device from numerous manufacturers. Price of EEG devices depends mainly on the number of electrode channels and performance. Cost of EEG device starts from $99 and can go beyond $25,000, which gives researchers buying flexibility.

Magnetoencephalography (MEG) uses magnetic potentials to record brain activity at the scalp level, using magnetic field sensitive detectors in the helmet placed on the subject’s head. Magnetic field is not influenced by the type of tissue (blood, brain matter, bones), unlike electrical field-based EEG, and can indicate the depth of the location in the brain with high spatial and temporal resolution [3]. Similar to MEG, transcranial magnetic stimulation (TMS) uses varying magnetic field [83] generated by electromagnetic induction using an iron core. TMS can stimulate targeted part of the brain, which enables it to conduct social or behavioral experiments. TMS and MEG are also used frequently in Neuromarketing experiments. However, the selected databases for this review did not contain any Neuromarketing research articles using these technologies over the last 5 years.

The electromyography (EMG) measures electrical activity produced by skeletal muscles when the muscles contracts and expands in order to move the body [70]. Also EMG is generated from the autonomic nervous activity related to emotional or mental activity. In Neuromarketing research, facial EMG is the best measure of the valence of the emotional reaction as it records facial muscle movement from two different muscles, i.e., zygomaticus muscle and corrugator muscle. Zygomatic muscle is found to react more while exposed to positive stimuli [70].

**Fig. 1:**
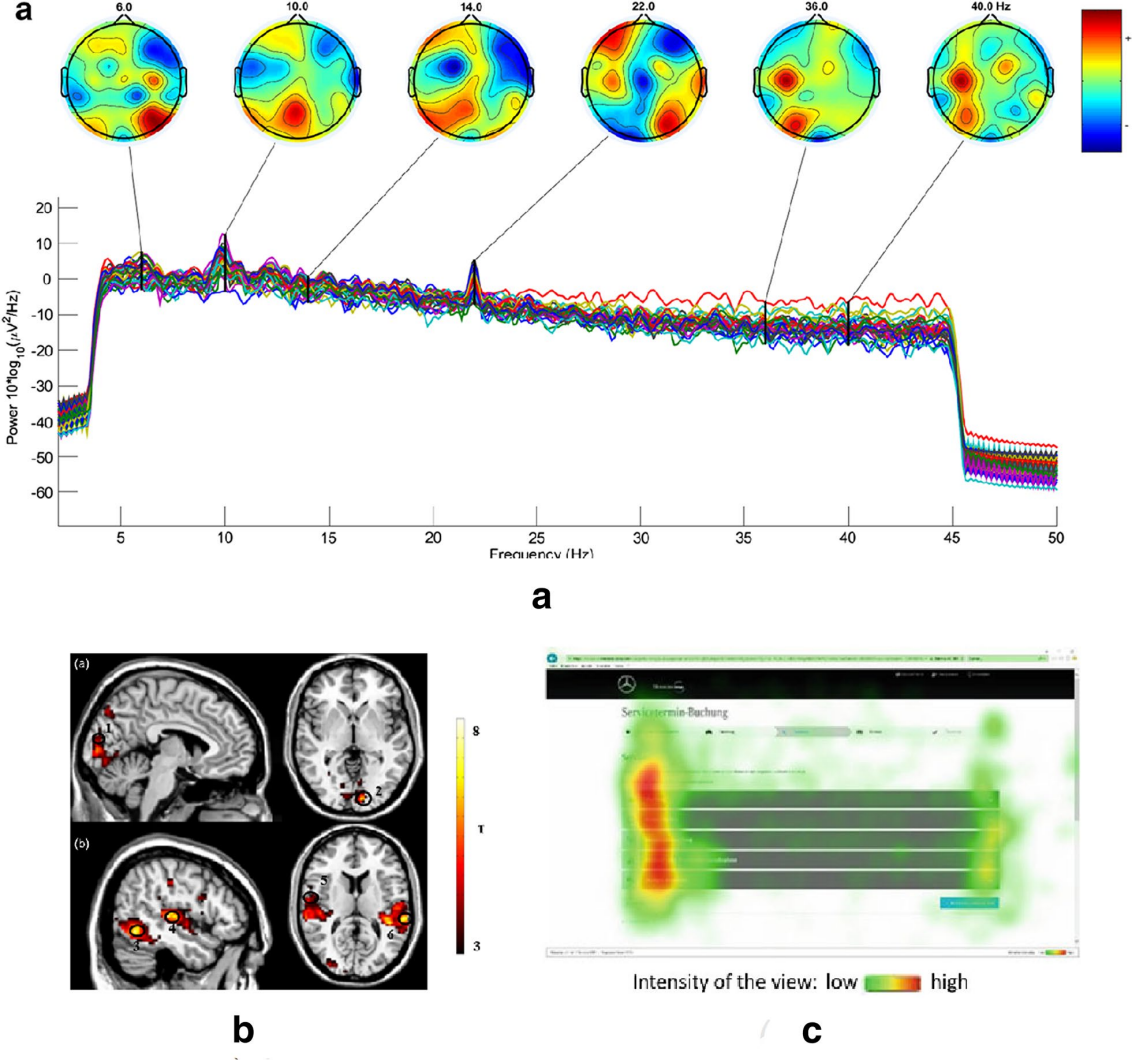
Neural recording in Neuromarketing experiments: **a** multichannel EEG [43], **b** fMRI imaging [50], and **c** eye tracking for online booking appointment [48]

Besides these brain signal recordings, eye tracking is the most popular method for analyzing consumer response. Eye tracking offers to measure visualization time and gaze path across a screen in Neuromarketing experiments. Besides tracking eye movement, pupil dilation measurement allows one to associate audience’s focus and arousal to the marketing stimuli. In the reviewed literatures, Tobii Pro X2-30 system from Tobii Technology was found as the most popular eye tracking device. In 2019, Etzold et al. used this eye tracking device to explore attention research on online booking [48]. Tobii Pro can also cooperate with fMRI-based Neuromarketing experiment (Venkatraman [38]). Other than Tobii, Eye Tribe is found in use by Çakar et al. [34]. Ungureanu et al. used eye tracking to measure the attention level of consumers while displaying static advertisements of cars and clothing products [53]. Figure 1 depicts the most popular methods of neural response recording i.e. EEG, fMRI and eye tracking used in the Neuromarketing experiments.

Some of the Neuromarketing studies used heart rate, as one of the metrics to measure arousal and focus of the consumer while they encounter TV commercial stimuli. Heart rate is the speed of the heartbeat and it is typically measured by electrocardiogram (EKG). An EKG measures the electrical activity of the heart using external skin electrodes. Heart rate is controlled by two antagonistic nervous systems, i.e., the sympathetic nervous system (SNS) and the parasympathetic nervous system (PNS). Automatic response to external stimuli is determined by the sympathetic system of the body. Activation of this system increases heart rate, causing fight or flight mode, which is an independent measure of arousal [38]. In contrast, the calm and relaxed state characterized by slower heart rate is controlled by the parasympathetic system. Slower heart rate in response to an advertisement implies the increased focus on the ad, hence provides an independent measure of attention [38]. Another physiological parameter, skin conductance (SC), or galvanic skin response (GSR), develops when the skin acts as an electrical conductor due to the increased activity of the sweat glands from exposure to stimulus [38]. Skin conductance amplitude and response latency provide direct measures of arousal when watching TV commercials, unlike self-reported measures that are often based on later memory recall. Although GSR cannot independently relate to emotional valence, some of the Neuromarketing studies, i.e., Cherubino et al. [42], Çakar et al. [34], Ungureanu et al. [53], Magdin et al. [71], Goyal and Singh [54], and Singh et al. [56] have used skin conductance along with heart rate to measure the consumer attention and focus on the TVC.

### Brain signal processing in Neuromarketing

Since neural signals and images are highly vulnerable to noise and artifacts, before performing any analysis or interpretation it is imperative to preprocess the neural signals to increase the signal-to-noise ratio (SNR). Noises that commonly accompany the EEG signals are cardiac signals (ECG), power line interference, eye movement artifact (EOG) and muscle movement artifacts (EMG). Preprocessing in Neuromarketing consists of filtering the signals to the frequency bands of interest, re-referencing the filtered signal to a common average, detecting and interpolating bad channels, noise and artifact removal, and framing or segmentation for further machine learning process.

EEG signals usually spread across its energy from 0.5 Hz to around 90 Hz. For classification purpose, it is required to have energies only from the relevant frequency bands, hence EEG preprocessing commonly uses band pass filtering techniques. Band pass filter requires two cutoff frequencies, one upper and one lower to pass the energy between them and blocks energies from all other frequencies. Band pass filter used in these Neuromarketing experiments are basically the digital version of the filter mostly applied by MATLAB and EEGLAB (a toolbox designated for EEG signal processing in MATLAB). Re-referencing to a common average reference is also found common after band pass filtering in the studies of Yang et al. [41], Fan and Touyama [66] to reduce possible shifts from external artifacts. Power line interference is usually found removed by using a notch filter at 60 Hz or 50 Hz.

The reviewed literatures had some common approaches in noise removal techniques. Since the noise accompanied with EEG signals are random in nature, signal averaging is a common approach to reduce these noises. Fan and Touyama [66] averaged the ERP signals for noise removal. Chew et al. [17] used ABM software development kit (SDK) in MATLAB to remove 5 types of artifacts, namely EMG, eye blinking artifact, excursions, saturations and spike. Excursion, saturation and spike artifacts in the EEG signals are replaced by zero values. Then they applied nearest neighbor interpolation to replace those zero values. Another type of filter Savitzky–Golay is found in use by Yadava et al. [18] for signal smoothing. For noise and artifact removal, the 4^th^-order Butterworth filter was used in the studies of Ogino and Mitsukura [68] and Oon et al. [55].

Independent component analysis (ICA) is an approach to separate the statistical subcomponents of EEG signals. ICA is found as the most sought after technique for removing artifacts and noise from EEG signals in these articles. Studies of Cherubino et al. [42], Bhardwaj et al. [53], Venkatraman et al. [38], Pozharliev et al. [20], Boksem and Smitds[57], Wriessnegger et al. [29], Fan and Touyama [66], Pilelienė and Grigaliūnaitė [36] all used independent component analysis mostly for eye blink and eye movement artifact, and muscular movement noise removal.

Neuromarketing with fMRI studies have a different method for image preprocessing. Since the fMRI provides a 3D image of the brain region with time information, it is basically a 4D signal. A 4D dataset is motion corrected for any head movement, slice time corrected, spatially normalized and finally smoothed to recover a denoised fMRI image. Wang et al. [30] used statistical parametric mapping (SPM) software to preprocess their fMRI data. Their raw fMRI signal was subjected to standard preprocessing involving correction for head motion, slice timing correction, temporal and spatial denoising and normalization into standardized Montreal Neurological Institute (MNI) space. The mean fMRI signal from each region of interest was extracted from voxels in a sphere of 6-mm radius centered at the activation point in the regional activation map.

fMRI scan was also used by Hubert et al. [25] in their experiment on hedonic vs. prudent shopper based on consumer impulsiveness. Decision-making process with cognitive deliberation and the consideration of long-term consequences are associated with processing in brain areas such as the ventromedial prefrontal cortex (vmPFC) and the dorsolateral prefrontal cortex (dlPFC). Hence, these vmPFC and dlPFC were the region of interests to capture the BOLD activation imaging [62]. Brain activation through BOLD signals was used by Hsu and Cheng [26] to investigate negative emotion after product harm crisis. fMRI region of interest in this study included amygdala, left calcarine, striatum, ventral tegmental area (VTA) and right insula. The amygdala is associated with memory and subjective evaluation, left calcarine relates to human visual processing, the striatum is associated with goal-oriented evaluation, and reward evaluation, VTA relates to decision-making process and motive functions, and the insula regions are involved in consumer decision-making related to negative reinforcement. Acquiring activation within these regions affirms the relation between stimuli and cognitive response.

Signal detection and segmentation is the process by which the signal of interest is detected from the original signal and then separated for further procedures. The energy of the signal may be used as a threshold for detection of the signal. Often the Neuromarketing experiments contain multiple types of stimuli shown to the test subjects. In such cases segmentation separates the event-based time signals for further processing, example Bhardwaj et al. [58]. Segmentation or framing the EEG signals to a shorter time window is mostly required to process the signal in time–frequency domain [58]. Cherubino et al. [42] segmented their acquired and filtered EEG traces to extract the cerebral activity during the exposure to the marketing stimuli. Oon et al. [55] used 1-s segmentation time to extract non-linear detrended fluctuation analysis features.

The goal of feature extraction is to find the set of feature that minimizes intra-class variability and maximizes inter-class variability. So we need to extract useful information from the preprocessed signal, which can be spatial, spectral or temporal [45]. As the EEG signal is non-stationary, the feature extraction procedure is quite often complicated. Discrete wavelet transformation (DWT) is a viable way to extract features from EEG signals.

Yadava et al. [18] performed DWT-based four-level wavelet analysis to extract features from their EEG signals and decomposed the EEG signal into delta, theta, alpha, beta and gamma frequency bands. Another feature extraction approach, principal component analysis (PCA) was used by Venkatraman et al. [38] for extracting fMRI features in their Neuromarketing experiment. In 2016, Fan and Touyama applied spatial and temporal principal component analysis (STPCA) for feature extraction from ERP P300 signal. Rakshit and Lahiri [67] used a different approach to extract features from EEG signals. They used Welch method for one-sided power spectral density estimate and then applied a 256-point DFT algorithm on hamming window of length 50 to extract features. Chew et al. [17] adopted Hadjidimitriou and Hadjileontiadis methods in feature extraction where the feature estimation is based on the event-related synchronization and desynchronization theory.

Feature selection is also popularly known as dimensionality reduction or subset selection. This is a well-known concept in machine learning which is about selecting an optimal set of features that decreases dimensionality, but has the most contribution to the classification accuracy. In the past few years, feature selection has caught the attention of most researchers because of the nature of high dimensionality of bio-signals and the low number of sample data. Selection of the optimal feature subset is always relative to an evaluation function. In most cases it is the evaluation function that measures the classification accuracy. Feature selection techniques can be divided into three categories, namely: filter, wrapper and embedded approach. Wang et al. [30] used Recursive Cluster Elimination (RCE) algorithm in spatiotemporal fMRI feature selection. Soria Morillo et al. [40] used PCA for feature reduction from their dataset. One-way analyses of variance (ANOVA) then cross-validation were also found in use to identify the optimal feature set for cognitive or affective state classification by Yang et al. [41].

### Machine learning application in Neuromarketing

Using advanced neural recording method and signal processing tools, one can analyze EEG signals and interpret their correspondence with marketing stimuli. Frontal alpha asymmetry theory helped the researcher classify emotional approach/withdrawal response of the test subjects using sub-band power of left and right hemispheric frontal electrode [21]. However, classifying approach/withdrawal or like/dislike without the FAA is possible, even possible from single electrode EEG signals. This requires advanced Machine Learning algorithm application in Neuromarketing. Both supervised and unsupervised learning methods were used in the following Neuromarketing experiments. Supervised learning in Neuromarketing uses a priori ground truth, usually the interviewed response (like/dislike) from the test subjects as the labels. The labels help the classifier know the signal pattern of like and dislike EEGs in the training datasets. During the testing phase, like/dislike is predicted from a dataset without the labels. Researcher can hide the training dataset labels from the classifier, and later use it for accuracy calculation. On the other hand, unsupervised learning approach used in Neuromarketing does not require prior knowledge of the like/dislike labels. It analyzes the signals with an aim to infer the existing structures for different classes. Supervised learning usually solves either classification problem or a regression problem. Support Vector Machines (SVM), Naive Bayes, Artificial Neural Networks (ANN), and Random Forests (RF) are the most common supervised learning classifiers in Neuromarketing. In parallel, unsupervised learning in Neuromarketing has prominently the clustering type classifiers, such as K-NN (k-nearest neighbors), principal component analysis, singular value decomposition, and independent component analysis (ICA).

Neuromarketing researches over the last 5 years mainly dealt with like/dislike classification problem and predicting consumer choice problem. Besides the learning method, both linear and non-linear classifiers have been used in these Neuromarketing experiments. The most used classification algorithms used in Neuromarketing over the last 5 years are Support Vector Machine (SVM), Linear Discriminant Analysis (LDA), Artificial Neural Network (ANN), Naïve Bayes, k-Nearest Neighbor (KNN) and Hidden Markov Model (HMM).

SVM is a supervised learning method, which requires training data for inferring a relation and recognizing patterns. SVM works as a discriminative classifier while a hyperplane separates the different classes. Based on the training data SVM creates a hyperplane which further classifies the new data. The advantage of using SVM in Neuromarketing is its computational simplicity and accuracy level. LDA classifiers are used in several literatures in comparison with SVM classifiers. LDA gathers data points with similar frequencies as distinct groups and 1D Eigen transformation creates the separate classes. Bhardwaj et al. [53] extracted energy and power spectral density as the feature from the acquired EEG signal and applied SVM and LDA classifiers to classify human emotions from EEG signals. Their model achieved 74.13% average accuracy for SVM-based emotion (happy, sad, anger, disgust, neutral, fear and surprised) classification. In contrast, the model achieved 66.50% average accuracy for LDA-based emotion classification. In the P300 signal-based experiment of Fan and Toyuyama, they used LD classifier to retrieve emotional faces from different subjects.

In 2016, Ogino and Mitsukura experimented on a single-channel EEG device for emotion estimation for mobile application. Their study used SVM, LR, KNN and SVR together to create a model of valence estimation from EEG signals. They used two regression methods linear regression (LR) and support vector regression (SVR) to define valence as sequential value from 1 to 9. SVM and KNN classified nine emotional classes, and SVR minimized the number of sample errors. Rakshit and Lahiri used SVM and interval-type 2 fuzzy classifiers to classify red blue and green colors from EEG signals. Their model achieved the classification with 78.81% average accuracy for SVM-based color classification [67]. However, IT2FS achieved the highest 80.04% mean accuracy compared to other classifiers in the experiment.

The hidden Markov model (HMM) is non-linear classifier under another supervised learning method. It is derived from statistical modeling and is widely used in temporal and biomedical signals. In Neuromarketing experiments, HMM is used to classify multiclass sequential data where transition from one mental state to another mental state can occur. Researchers can find possible observation of the states using the state transition probabilities. Yadava et al. proposed an HMM-based consumer choice prediction (like/dislike) model using EEG signals from frontal, parietal, temporal and occipital lobe. They compared their classification model with standard classifiers such as SVM, RF and ANN. Their HMM-based model achieved classification accuracy of 70.33% for male test subjects and 63.56% for female test subjects [18]. In comparison, accuracy of 62.85% was achieved with SVM classifier with *C* = 6, whereas ANN with two hidden layers achieved 60% average accuracy.

K-Nearest Neighbor algorithm serves both as a classification and regression algorithm. KNN algorithm predicts the test sample’s category based on to the K training samples which are the nearest neighbors to the test sample. In contrast to the hyperplane of SVM, KNN creates a decision boundary among different distinct classes. In the experiment of Chew et al. [17], SVM and KNN are used to explore the esthetic preference for 3D shapes. The mean accuracy for SVM classifier obtained was 68%, whereas the mean accuracy for KNN classifier was 64%.

Artificial Neural Network (ANN) is a form of neural network classifiers. ANN is a collection of artificial neurons which produces non-linear decision boundaries among large number of classes. ANN and its different subtypes are now becoming more common for the Neuromarketing data interpretation. However, ANN requires large number of sample data and large number of features. Soria Morillo et al. used ANN algorithm in 2015 and 2016 in comparison with Random Forest algorithm C4.5 and Ameva, respectively. In 2015, their advertisement liking recognition model achieved 80% average accuracy with ANN and 69.4% for C4.5 classifier [43]. In 2016, ANN, C4.5 and Ameva achieved average accuracy of 80%, 69%, and 75%, respectively.

Oon et al. focused on recognizing preference among different categories of products (food, automobile, etc.) using KNN and ANN to analyze non-linear features of the EEG signals [55]. ANN and KNN inputs were used as the features for Detrended Fluctuation Analysis (DFA) which achieved the highest classification accuracy 80% for alpha waves, and 76.18% for beta waves. Doborjeh et al. [64] used another type of Neural Network, Spiking Neural Network (SNN) to recognize attention bias pattern from spatio-temporal EEG signal. In their study, a brain-like SNN methodology (NeuCube) was used to create models from EEG signals to evaluate how attention bias can affect the consumer preferences. Their SNN-based classification model achieved 89.95% average accuracy, while traditional machine learning SVM classifier achieved 48.5% accuracy.

## Result synthesis

This section synthesizes the results from already discussed research articles and book chapters with empirical findings on Neuromarketing, published from 2015 to 2019. To ensure the reliability of the experimental findings, the reviewed literatures had largely set their statistical significance at *p* < 0.05 [20, 38, 42, 43, 46, 59, 60, 70].

With the advancements in technologies, marketing stimuli have become more TV commercial or image of the product oriented rather than the original product [18–26, 34–43]. 3D image of the products have also added to these virtual product purchase decision-making [17]. E-commerce products have gained interest among the Neuromarketing researchers, since these products are now more available to the consumers through online shopping [34]. First-time user experience in online shopping and user experience in online appointment have also diversified the stimuli group of Neuromarketing research. Other than these marketing focused stimuli, some of the Neuromarketing studies focused on social advertisements, particularly the campaign against smoking and alcohol consumption among young adults. These social advertisements used neuroimaging and neural signal decoding techniques to assess and predict the success of their message reaching the targeted social groups.

Analyzing consumer’s emotional response is found as a focus of current Neuromarketing research articles. These experiments widely used Frontal Alpha Asymmetry theory for left and right frontal channel. Besides the alpha band, beta and theta bands are also found in use in these literatures to recognize cognitive and emotional response of the consumers. Table 3 summarizes the findings related to brainwaves and their functionalities in the reviewed Neuromarketing literatures.

**Table 3:** Functionalities of brain states used in Neuromarketing research

Brain states	Functionalities in Neuromarketing
Theta (4–8 Hz)	Frontal theta associated with cognitive process [59]. Theta amplitude increase for preferred color [18].
Alpha (8–12 Hz)	Frontal alpha associated with cognitive process [59]. Alpha amplitude is inversely correlated with neural activity used in frontal asymmetry score [21]. Emotional valance corresponds alpha asymmetry, high alpha activity in central–parietal–occipital lobe vigilance [27].
Beta (12–30 Hz)	Medial–frontal beta band activity is associated with reward processing [57]. Right parietal beta corresponds to imagination [59].

Over the last 5 years in consumer neuroscience research, the use of research-grade commercially available EEG devices has become more popular than fMRI scanners. EEG has been particularly used in TV advertisement evaluation, where a high temporal resolution is required to explore the dynamic effects of TV commercials. Even though fMRI has been used less in the Neuromarketing experiments, the use of fMRI is particularly found when a consumer is displayed product images and asked to make purchase decision [30]. The reason behind using product images as marketing stimuli in fMRI-based Neuromarketing research is that, fMRI can point out the activated brain region when a subject encounters a marketing stimuli. The activated brain region can estimate the positive or negative experience of the consumer in their brain. However, TVC changes stimuli in millisecond time frame, response of which cannot be obtained by an fMRI scanner with 2–5 s image refresh rate. Other than EEG and fMRI, fNIRS has started to enter the Neuromarketing research field. Having the advantage of mobility, fNIRS has been used in purchase behavior correlation and consumer reaction examination by Çakir et al. and Krampe et al. In these cases, fNIRS has shown accuracy over 70% and scored in reliability scale 0.7 out of 1, respectively [24, 60]. This shows fNIRS can be a promising mean of neural recording for future Neuromarketing experiments.

While comparing the EEG devices, Emotiv Epoc and Emotive Epoc+ had the largest number of academic research conducted through them. Other than the 14-channel device, BrainAmp is a 10-channel EEG device and eego Sports is a 32-channel device used by Neuromarketing researchers. NeuroSky MindWave despite having only one sensor, provided denoised EEG data and performed well with accuracy over 70%.

All of the fMRI-based Neuromarketing studies over the last 5 years have used 3-Tesla fMRI scanner Magnetom Trio, SIEMENS, and Siemens Verio scanner for their experiments [25, 30, 62]. The advantage of 3.0-T functional MRI is the high spatial resolution. However, BOLD signal-based fMRI has the possible confusion with blood flow due to head or muscle movement.

Signal preprocessing in the selected articles was mainly performed by using MATLAB and EEGLAB. Besides band pass filtering, increased used of independent component analysis (ICA) in spatiotemporal domain is also observed over the course of last 5 years [20, 36, 38, 42, 53]. Other than noise and artifact removal, preprocessing dealt with framing or segmentation of the temporal EEG signal. The fMRI data were preprocessed using the statistical parametric mapping (SPM) software.

In this systematic review, a number of Neuromarketing research experiments used artificial intelligent algorithms for prediction and classification purposes. Table 4 compares the average classification accuracy achieved by these algorithms in the selected Neuromarketing studies.

**Table 4:** Comparative accuracy analysis for machine learning classifiers in Neuromarketing

Classifiers	Neuromarketing studies	Average accuracy
Support Vector Machine (SVM)	Like/dislike classification for esthetic preference recognition among 3D objects (Chew et al.) [17]	68%
	Attention bias identification between targeted and non-targeted stimuli using NeoCube-based SNN architecture (Doborjeh et al.) [64]	48.5%
	Like/dislike classification among e-commerce product (Yadava et al.) [18]	62.85%
	Emotional valence recognition between excitement and boredom using EEG device and combining SVM, KNN, SVR, LR (Ogino and Mitsukura) [68]	72.4%
	Purchase decision prediction from fMRI data using recursive cluster elimination-based support vector machine (RCE-SVM) (Wang et al.) [30]	55.70%
	Facial emotion recognition using GSR sensor biometric data (Goyal and Singh) [54]	81.65%
	Seven-emotion recognition using EEG signal (Bhardwaj et al.). Happiness and sadness classification accuracy reported here, respectively	87.5%, 92.5%
	Color classification using EEG signal (Rakshit et al.)	78.81%
K-Nearest Neighbor (KNN)	Like/dislike classification for esthetic preference recognition among 3D objects (Chew et al.) [17]	64%
Hidden Markov model (HMM)	Like/dislike classification among e-commerce product (Yadava et al.) [18]. Classification accuracy reported for male and female subject, respectively	70.33%, 63.56%
Linear discriminant analysis (LDA)	Seven-emotion recognition using EEG signal (Bhardwaj et al.) [58]. Happiness and sadness classification accuracy reported here, respectively	82.5, 87.5%
	Like-/dislike classification using car stimuli and ERP signal (Wreissenger et al.)	61%
Naïve Bayes	Purchase decision prediction using Neural Impulse Actuator (NIA) device (Taqwa et al.) [73]	48.5%
Artificial Neural Network	Consumer gender prediction using facial action coding (Gurbuj and Toga) [28]	83.8%
	TV advertisement liking recognition using EEG signal (Soria Morillo et al.) [43]	80%
	TV advertisement liking recognition using EEG (Soria Morillo et al.) [40]	80%
	Like/dislike classification among e-commerce products (Yadava et al.) [18]	60%

While comparing the classification performance of machine learning algorithms in Neuromarketing research, we found the Artificial Neural Network had the highest classification accuracy around 80% among all other algorithms [40, 43]. However, ANN requires more training data than other classifiers such as 70% data in training and 30% in testing, which calls into question its viability in Neuromarketing. After ANN, SVM was the algorithm most widely used in Neuromarketing with the second highest classification accuracy above 70%. HMM performed better than KNN in overall application of machine learning algorithms in Neuromarketing.

## Recommendation

From this systematic review, authors would like to suggest future Neuromarketing researchers to first define the scope of their inquisition, which defines the rest of the process. Neuromarketing on product purchase assessment and purchase decision-making have been using functional MRI to locate the activated region in consumer brain to predict the success or failure of the product. However, to recognize consumer engagement with product commercial, it is worthwhile to use EEG devices with high temporal resolution. Neuromarketing experiments with EEG devices of 14 channels and 32 channels have established their research-grade performance. However, the raw data availability should be kept in mind by the researchers while selecting an EEG device. Also, researcher should consider availability of bilateral EEG electrodes if they would like to utilize frontal alpha asymmetry theory. Accompanying EEG, eye tracking has also shown high performance in attention and arousal locating. Eye tracker, heart rate monitor, galvanic skin response device can be used alongside brain signal to cross-validate the experimental findings. While choosing among classifiers, although ANN has shown better performance consistently. However, authors would recommend preferring linear classifier over neural networks, as most of the Neuromarketing sampling EEG dataset does not contain plethora of samples to train a complex classifier as ANN.

## Conclusion

Neuromarketing is an emerging field with opportunities in commercial, social and political advertisement domain. The advancements of this field hence requires proper documentation to capture its state-of-art. This study was conducted with a focus to shed light on the technological scope and possible opportunities in this field. Authors found over the course of last 5 years, Neuromarketing experiments have been conducted mainly with the stimuli of consumer goods, in both product and promotion forms. However, Neuromarketing is showing its possibilities in the domain of social advertisement. Neuromarketing researchers tend to focus on the frontal and prefrontal cortex of consumer brain for cognitive and emotional inquiries. Among all brain signal recording devices, we found EEG is becoming more popular in Neuromarketing experiments, especially with TVC analysis due to its high temporal resolution and cost effectiveness. However, EEG devices have different sampling rates causing a limitation for highest analyzable frequency, which should be under the scrutiny of the researchers. Signal processing in these studies largely adopted ICA for noise and artifact removal. Finally, the highest number of studies have used SVM for classification purpose among all other algorithms, perhaps due to its simplicity. We hope, our findings will guide future researchers to explore the opportunities in this field in a more efficient manner.

## Data Availability

This review used available literature relevant to the problem statement from valid databases across the internet. Databases are: Science Direct, Emerald Insight, Sage, IEEE Xplore, Wiley Online Library, and Taylor Francis Online.

## Abbreviations

ANN: Artificial Neural Network
DWT: Discrete wavelet transformation
DFA: Detrended fluctuation analysis
EEG: Electroencephalography
fMRI: Functional magnetic resonance imaging
fNIRS: Functional near infra-red spectroscopy
GSR: Galvanic skin response
HMM: Hidden Markov model
HR: Heart rate
ICA: Independent component analysis
KNN: K-Nearest Neighbor
LDA: Linear discriminant analysis
MEG: Magneto encephalography
PCA: Principal component analysis
PFC: Prefrontal cortex
SVM: Support Vector Machine
TVC: TV commercial

## References

[1] Assael H (1981) Consumer behavior and marketing action

[2] Malhotra NK (1993) Marketing research: an applied orientation

[3] Vecchiato G, Astolfi L, Fallani FV (2011), On the Use of EEG or MEG brain imaging tools in neuromarketing research, computational intelligence and neuroscience 2011, Article ID 643489 10.1155/2011/643489 PMC3180786 21960996

[4] Izhikevich EM (2003). Simple model of spiking neurons. IEEE Transac Neural Netw..

[5] Custdio PF (2010) Use of EEG as a neuroscientific approach to advertising research, Master thesis, Instituto Superior Tcnico, Universidade Tecnica De Lisboa

[6] Dimpfel W (2015). Neuromarketing: neurocode-tracking in combination with eye-tracking for quantitative objective assessment of TV commercials. J Behav Brain Sci..

[7] Kroupi E, Hanhart P, Lee JS, Rerabek M, Ebrahimi T (2014) Predicting subjective sensation of reality during multimedia consumption based on EEG and peripheral physiological signals. In: International conference on multimedia and expo, pp 1–6

[8] Rami NK, Chelsea W, Sarath K, Jordan L, Barbara EK (2013). Consumer neuroscience: assessing the brain response to marketing stimuli using electroencephalogram (EEG) and eye tracking. Expert Syst Appl.

[9] Ariely D, Berns GS (2010). Neuromarketing: the hope and hype of neuroimaging in business. Nat Rev Neurosci.

[10] Sing D, Sharma JK (2010), Neuromarketing: a peep into customer S minds

[11] Neuromarketing Science and Business Association (NMSBA), The Global Neuromarketing Network, https://www.nmsba.com/. Accessed 28 July 2019

[12] Neuromarketing World Forum, http://neuromarketingworldforum.com/. Accessed 19 Oct 2019

[13] Cruz ML, Marcon A, Medeiros JF (2016). Neuromarketing and the advances in the consumer behaviour studies: a systematic review of the literature. Int J Bus Glob.

[14] Hsu M (2017). Neuromarketing: inside the mind of the consumer. Calif Manag Rev.

[15] Shaw SD, Bagozzi RP (2018). The neuropsychology of consumer behavior and marketing. Consum Psychol Rev..

[16] Khan KS, Kunz R, Kleijnen J, Antes G (2003). Five steps to conducting a systematic review. J R Soc Med.

[17] Chew LH, Teo J, Mountstephens J (2015). Aesthetic preference recognition of 3D shapes using EEG. Cognit Neurodynamics..

[18] Yadava M, Kumar P, Saini R, Roy PP, Dogra DP (2017). Analysis of EEG signals and its application to neuromarketing. Multimedia Tools Appl..

[19] Rojas JC, Contero M, Bartomeu N, Guixeres J (2015). Using combined bipolar semantic scales and eye-tracking metrics to compare consumer perception of real and virtual bottles. Packag Technol Sci..

[20] Pozharliev R, Verbeke WJMI, Van Strien JW, Bagozzi RP (2015). Merely being with you increases my attention to luxury products: using EEG to understand consumers’ emotional experience with luxury branded products. J Mark Res.

[21] Touchette B, Lee SE (2016). Measuring neural responses to apparel product attractiveness: an application of frontal asymmetry theory. Cloth Text Res J.

[22] Marques JP, Martins M, Ferreira HA, Ramalh J, Seixas D (2016), Neural imprints of national brands versus own-label brands, J Prod Brand Manage, 25(2)

[23] Shen Y, Shan W, Luan J (2018). Influence of aggregated ratings on purchase decisions: an event-related potential study. Eur J Mark.

[24] Çakir MP, Çakar T, Girisken Y, Yurdakul D (2018). An investigation of the neural correlates of purchase behavior through fNIRS. Eur J Mark.

[25] Hubert M, Linzmajer M, Riedl R, Kenning P (2018). Trust me if you can—neurophysiological insights on the influence of consumer impulsiveness on trustworthiness evaluations in online settings. Eur J Mark.

[26] Hsu L, Chen Y (2019). Music and wine tasting: an experimental neuromarketing study. Br Food J.

[27] Hoefer D, Handel M, Mueller K, Hammer TR (2016). Electroencephalographic study showing that tactile stimulation by fabrics of different qualities elicit graded event-related potentials. Skin Res Technol.

[28] Gurbuz F and Toğa G, Usage Of The Facial Action Coding System To Predict Customer Gender Profile: A Neuro Marketing Application In TURKEY. 2018 2nd International Symposium on Multidisciplinary Studies and Innovative Technologies (ISMSIT) (2018): 1–4

[29] Wriessnegger S.C., Hackhofer D., Müller-Putz G.R. (2015), Classification of unconscious like/dislike decisions: First results towards a novel application for BCI technology Conference Proc IEEE Eng Med Biol Soc. 2015;2015:2331–4. doi: 10.1109/EMBC.2015.7318860. 10.1109/EMBC.2015.7318860 26736760

[30] Wang Y, Chattaraman V, Kim H, Deshpande G (2015). Predicting purchase decisions based on spatiotemporal functional MRI features using machine learning. IEEE Trans Auton Ment Dev.

[31] Wolfe K, Jo W, Olds D, Asperin A, DeSanto J, Liu WC (2016). An fMRI study of the effects of food familiarity and labeling on brain activation. J Culi Sci Technol.

[32] Bosshard SS, Bourke JD, Kunaharan S, Koller M, Walla P (2016). Established liked versus disliked brands: brain activity, implicit associations and explicit responses. Cogent Psychol.

[33] Fehse K, Simmank F, Gutyrchik E, Sztrókay-Gaul A (2017). Organic or popular brands—food perception engages distinct functional pathways. An fMRI study. Cogent Psychol.

[34] Çakar T, Rızvanoğlu K, Öztürk O, Çelik DZ, and Gürvardar I (2017) The use of neurometric and biometric research methods in understanding the user experience during product search of first-time buyers in e-commerce, international conference of design, user experience, and usability

[35] Gong Y, Hou Z, Zhang Q, Tian S (2018). Discounts or gifts? Not just to save money: a study on neural mechanism from the perspective of fuzzy decision. J Contemp Market Sci.

[36] Pilelienė L and Grigaliūnaitė V, (2017), The effect of female celebrity spokesperson in FMCG advertising: neuromarketing approach, J Consum Market, 34(3)

[37] Boccia F, Malgeri Manzo R, Covino D (2019). Consumer behavior and corporate social responsibility: an evaluation by a choice experiment. Corp Soc Resp Env Ma..

[38] Venkatraman V, Dimoka A, Pavlou PA, Vo K, Hampton W, Bollinger B, Hershfield HE, Ishihara M, Winer RS (2015). Predicting advertising success beyond traditional measures: new insights from neurophysiological methods and market response modeling. J Market Res.

[39] Baldo D, Parikh H, Piu Y, Müller KM (2015). Brain waves predict success of new fashion products: a practical application for the footwear retailing industry. J Creat Val.

[40] Soria Morillo LM, Álvarez-García JA, Gonzalez-Abril L, Ramirez JA (2015) Advertising liking recognition technique applied to neuromarketing by using low-cost EEG Headset. IWBBIO 10.1186/s12938-016-0181-2 PMC4959374 27454876

[41] Yang T, Lee DY, Kwak Y, Choi J, Kim C, Kim SP (2015). Evaluation of TV commercials using neurophysiological responses. J Physiol Anthropol..

[42] Cherubino P, Trettel A, Cartocci G, Rossi D, Modica E, Maglione AG, Mancini M, Flumeri GD, Babiloni F (2016) Neuroelectrical indexes for the study of the efficacy of TV advertising stimuli

[43] Soria Morillo LM, Álvarez-García JA, Gonzalez-Abril L (2016). Ramirez JA (2016) Discrete classification technique applied to TV advertisements liking recognition system based on low-cost EEG headsets. Biomed Eng Online..

[44] Vasiljević T, Bogdanović Z, Rodić B, Naumović T, Labus A, Rocha Á, Adeli H, Reis L, Costanzo S (2019). Designing IoT infrastructure for neuromarketing research. New knowledge in information systems and technologies. WorldCIST’19 2019. Advances in Intelligent Systems and Computing.

[45] Yang D (2018). Exploratory neural reactions to framed advertisement messages of smoking cessation. Soc Market Quart.

[46] Daugherty T, Hoffman E, Kennedy K, Nolan M (2018). Measuring consumer neural activation to differentiate cognitive processing of advertising: revisiting Krugman. Eur J Mark.

[47] Royo M, Chulvi V, Mulet E, Galán J (2018). Users’ reactions captured by means of an EEG headset on viewing the presentation of sustainable designs using verbal narrative. Eur J Mark.

[48] Etzold VM, Braun A, Wanner T (2019) Eye tracking as a method of neuromarketing for attention research—an empirical analysis using the online appointment booking platform from Mercedes-Benz

[49] Chen Y, Fowler CH, Papa VB, Lepping RJ, Brucks MG, Fox AT, Martin LE (2018). Adolescents’ behavioral and neural responses to e-cigarette advertising. Addict Biol.

[50] Casado-Aranda L, Laan LN, Sánchez-Fernández J (2018). Neural correlates of gender congruence in audiovisual commercials for gender-targeted products: an fMRI study. Hum Brain Mapp.

[51] Randolph, A.B., & Pierquet, S. (2015). Bringing advertising closer to mind: using neurophysiological tools to understand student responses to super bowl commercials. 2015 48th Hawaii International Conference on System Sciences, 517–522

[52] Nomura T and Mitsukura Y (2015), Extraction of unconscious emotions while watching TV commercials IECON 2015—41st Annual Conference of the IEEE Industrial Electronics Society, art. no. 7392127, pp. 368–373

[53] Ungureanu F, Lupu RG, Cadar A, Prodan A (2017) Neuromarketing and visual attention study using eye tracking techniques, 21st International Conference on System Theory, Control and Computing (ICSTCC)

[54] Goyal G and Singh J (2018), Minimum Annotation identification of facial affects for Video Advertisement, International Conference on Intelligent Circuits and Systems

[55] Oon HN, Saidatul A, Ibrahim Z. et al. (2018), Analysis on Non-linear features of electroencephalogram (EEG) signal for neuromarketing application, 2015 48th Hawaii International Conference on System Sciences

[56] Singh J, Goyal G, Gill R (2019). Use of neurometrics to choose optimal advertisement method for omnichannel business. Enterprise Inform Syst.

[57] Boksem M, Smitds A (2015). Brain responses to movie trailers predict individual preferences for movies and their population-wide commercial success. J Market Res.

[58] Bhardwaj A, Gupta A, Jain P, Rani A, Yadav J (2015). Classification of human emotions from EEG signals using SVM and LDA Classifiers. 2015 2nd International Conference on Signal Processing and Integrated Networks (SPIN), 180–185

[59] Gordon R, Ciorciari J, Laer TV (2018). Using EEG to examine the role of attention, working memory, emotion, and imagination in narrative transportation. Eur J Mark.

[60] Krampe C, Strelow E, Haas A, Kenning P (2018). The application of mobile fNIRS to shopper neuroscience–first insights from a merchandising communication study. Eur J Mark.

[61] Holst EMZ, Henseler J (2017). Thinking outside the box: a neuroscientific perspective on trust in B2B relationships. IMP J.

[62] Hsu YT, Cheng MS (2018). fMRI neuromarketing and consumer learning theory: word-of-mouth effectiveness after product harm crisis. Eur J Mark.

[63] Anysha Jain, Tanupriya Choudhury, Ruby Singh, Praveen Kumar, (2018), Signal classification for real-time neuro marketing applications, International Conference on advances in computing and communication engineering (ICACCE-2018)

[64] Gholami Doborjeh Z, Doborjeh MG, Kasabov N (2018). Attentional bias pattern recognition in spiking neural networks from spatio-temporal EEG. Cogn Comput.

[65] Kaur B., Singh D., Roy P.P. (2018) Eyes Open and Eyes Close Activity Recognition Using EEG Signals. In: Nagabhushan T., Aradhya V., Jagadeesh P., Shukla S., M.L. C. (eds) Cognitive Computing and Information Processing. CCIP 2017. Communications in Computer and Information Science, vol 801. Springer, Singapore

[66] Fan J and Touyama H (2016), Emotional Face Retrieval with P300 signals of multiple subjects, joint 8th International Conference on Soft Computing and Intelligent Systems and 17th International Symposium. on Advanced Intelligent Systems

[67] Rakshit A and Lahiri R(2016), Discriminating different color from EEG signals using interval-type 2 fuzzy space classifier (a neuro-marketing study on the effect of color to Cognitive State), 1st IEEE International Conference on Power Electronics; Intelligent Control and Energy Systems (ICPEICES-2016)

[68] Ogino M, Mitsukura Y (2018), A mobile application for estimating emotional valence using a single-channel EEG device

[69] Missaglia A, Oppo A, Mauri M, Ghiringhelli B, Ciceri A, Russo V (2017). The impact of emotions on recall: An empirical study on social ads

[70] Ceravolo MG, Farina V, Fattobene L, Leonelli L, Raggetti GM (2019). Presentational format and financial consumers’ behaviour: an eye-tracking study. Int J Bank Market.

[71] Magdin M, Kohutek M, Koprda S, Balogh Z, (2019), EmoSens–the proposal of system for recognition of emotion with SDK affectiva and various sensors, in: intelligent computing theories and application

[72] Clerico A, Gupta R and Falk TH (2015), Mutual Information Between Inter-Hemispheric EEG Spectro-Temporal Patterns: A New Feature for Automated Affect Recognition, 7th Annual International IEEE EMBS Conference on Neural Engineering

[73] Taqwa T, Suhendra A, Hermita M, and Darmayantie A (2015), Implementation of Naïve Bayes method for product purchasing decision using neural impulse actuator in neuromarketing, International Conference on Information & Communication Technology and Systems (ICTS)

[74] Nemorin S (2016). Neuromarketing and the “poor in world” consumer: how the animalization of thinking underpins contemporary market research discourses. Consum Market Cult.

[75] Grönroos C (1990), “Marketing Redefined”, Management Decision, 28(8). 10.1108/00251749010139116

[76] MacLean PD (1988) Triune Brain. In: Comparative Neuroscience and Neurobiology. 126–128

[77] Nolte J, and Sundsten J (2009) The Human Brain: an Introduction to Its Functional Anatomy. Mosby/Elsevier

[78] Frackowiak S, Richard J (2007). Human brain function.

[79] Beeson P, Rapcsak S, Plante E, Chargualaf J, Chung A, Johnson S, Trouard T (2003). The neural substrates of writing: a functional magnetic resonance imaging study. Aphasiology.

[80] Vecchiato G, Toppi J, Astolfi L, Fallani FDV (2011), Spectral EEG frontal asymmetries correlate with the experienced pleasantness of TV commercial advertisements, Medical & Biological Engineering & Computing > Issue 5 10.1007/s11517-011-0747-x 21327841

[81] Abdullah-Al-Mamun, Khondaker (2013) Pattern identification of movement related states in biosignals. University of Southampton, Faculty of Engineering and the Environment, Doctoral Thesis, 225 pp

[82] Klem GH, Lüders H, Jasper HH, Elger C (1958). The ten-twenty electrode system of the International Federation. The International Federation of Clinical Neurophysiology. Electroencephalogr Clin Neurophysiol Suppl.

[83] Jolij J, Lamme VAF (2005). Repression of unconscious information by conscious processing: evidence from affective blindsight induced by transcranial magnetic stimulation. Proc Natl Acad Sci.

